# Relationship between the triglyceride glucose index and coronary artery calcification in Korean adults

**DOI:** 10.1186/s12933-017-0589-4

**Published:** 2017-08-23

**Authors:** Min Kyung Kim, Chul Woo Ahn, Shinae Kang, Ji Sun Nam, Kyung Rae Kim, Jong Suk Park

**Affiliations:** 1 Division of Endocrinology, Department of Internal Medicine, Hallym University Kangdong Sacred Heart Hospital, 150, Seongan-ro, Gangdong-gu, Seoul, South Korea; 2 Department of Internal Medicine, Gangnam Severance Hospital, Yonsei University College of Medicine, 211 Eonju-ro, Gangnam-gu, Seoul, South Korea; 3 Severance Institute for Vascular and Metabolic Research, Yonsei University College of Medicine, 211 Eonju-ro, Gangnam-gu, Seoul, South Korea

**Keywords:** TyG index, Coronary artery calcification, HOMA-IR, Atherosclerosis

## Abstract

**Background:**

The triglyceride glucose (TyG) index has been considered a simple surrogate marker of insulin resistance. However, few studies have investigated the relationship between the TyG index and coronary artery calcification (CAC). Thus, we investigated the relationship between the TyG index and CAC in healthy Korean adults.

**Methods:**

In total, 4319 participants who underwent cardiac computed tomography (CT) in a health promotion center were enrolled. Anthropometric profiles and multiple cardiovascular risk factors were measured. The TyG index was calculated as ln [fasting triglycerides (mg/dL) × fasting glucose (mg/dL)/2], and the insulin resistance index of homeostasis model assessment (HOMA-IR) was estimated. The CAC was measured using multidetector CT, and CAC presence was defined as an Agatston score of >0.

**Results:**

All subjects were stratified into four groups based on their TyG indices. Significant differences were observed in cardiovascular parameters among the groups, and the prevalence of CAC significantly increased with increasing TyG index. In the logistic regression analysis after adjustment for multiple risk factors, the odds ratio for the prevalence of CAC, when comparing the highest and lowest quartiles of the TyG index was 1.95 (95% CI 1.23–3.11; P for trend = 0.01); the odds ratio for the prevalence of CAC, when comparing the highest and lowest quartiles of HOMA-IR was 1.64 (95% CI 1.12–2.40; P for trend = 0.04). In the receiver operating characteristics analysis, the TyG index was superior to HOMA-IR in predicting CAC.

**Conclusion:**

The TyG index is more independently associated with the presence of coronary artery atherosclerosis than is HOMA-IR in healthy Korean adults.

**Electronic supplementary material:**

The online version of this article (doi:10.1186/s12933-017-0589-4) contains supplementary material, which is available to authorized users.

## Background

Cardiovascular disease (CVD) is the leading cause of morbidity and mortality worldwide. Coronary artery calcification (CAC), as determined by multidetector computed tomography (CT), is a sensitive measure for detecting the existence of early coronary artery atherosclerosis. Moreover, CAC can have prognostic value for predicting future CVD events [1–3].

Insulin resistance (IR) is associated with an increased risk of hyperglycemia, hypertension, and dyslipidemia, which increases the risk of inflammation, altered coagulation, and atherosclerosis. Many studies have demonstrated that IR is one of the most important contributing factors to CVD [4–6].

Recently, the triglyceride glucose (TyG) index has been proposed as a reliable and simple surrogate marker of IR. This index is correlated with IR, as assessed by the hyperinsulinemic euglycemic clamp test and the homeostatic model assessment of insulin resistance (HOMA-IR) [7–11].

Few studies have examined the relationship between the TyG index and CVD events, and their results have been inconsistent [12, 13]. In addition, no study has investigated the relationship between the TyG index and CAC in healthy adults.

Therefore, in the present study, we investigated the relationship between the TyG index and CAC and compared the index with HOMA-IR in healthy Korean adults.

## Methods

### Study population

The study population comprised 5217 Korean subjects who participated in a comprehensive health examination, including cardiac CT, as part of a self-referred health checkup program at the Gangnam Severance Hospital Health Promotion Center from January 2008 to February 2015 [14]. The exclusion criteria of this study included patients with elevated triglyceride levels (≥400 mg/dL), any malignancy, acute inflammatory disease, or infectious disease. We also excluded subjects with a history of angina, myocardial infarction or cerebrovascular accidents, hypertension, diabetes, or renal disease. Subjects taking statins or triglyceride-lowering medications (fenofibrate or omega-3) were also excluded. Ultimately, 4319 subjects were enrolled in our final analysis. The study protocol was approved by the Institutional Review Board of Yonsei University College of Medicine.

### Clinical characteristics

Height and weight were measured, and body mass index (BMI) was calculated by dividing the weight (kg) by the square of the height (m^2^). Lifestyle, personal medical history of acute or chronic illness, and medication history were assessed using a standard questionnaire. Systolic and diastolic blood pressures were measured by experienced technicians using an automated blood pressure (BP) monitor (HEM-7080IC; Omron Healthcare, Lake Forest, IL, USA) by placing the arm at heart level after a 5-min rest period. An individual who drinks at least 20 g/day of alcohol was defined as an alcohol drinker. Regular exercise was reported to be positive if the participant performed moderate exercise for more than 30 min at least three times a week. Current smokers were defined as those who reported having smoked cigarettes regularly over the previous 6 months.

### Biochemical parameters

Blood samples were obtained from all participants after 12 h of fasting. Fasting plasma glucose (FPG), total cholesterol (TC), high-density lipoprotein cholesterol (HDL-C), and triglyceride (TG) levels were determined by enzymatic methods using a Hitachi 7600-120 automated chemistry analyzer (Hitachi, Tokyo, Japan). The low-density lipoprotein cholesterol (LDL-C) level was calculated according to the Friedewald formula. The TyG index was calculated as ln[fasting triglycerides (mg/dL) × fasting glucose (mg/dL)/2] [7]. The fasting serum insulin level was determined using chemiluminescence (RIA kit, Daiichi, Japan), and the HOMA-IR index was calculated using the following formula: HOMA-IR = fasting insulin (µU/mL) × fasting glucose (mg/dL)/405.

### CAC measurements by multidetector CT

Coronary artery calcification was determined using a multidetector CT scanner (Philips Brilliance 64; Philips Medical System, Best, The Netherlands). A standard prospective ECG-gating protocol with a step-and-shoot technique was used, as previously described [15]. Coronary CT images were analyzed by one of three experienced radiologists, all of whom were blinded to the laboratory and clinical details of the participants at the time of analysis. The quantitative coronary artery calcification score (CACS) was calculated using dedicated software and was expressed as an Agatston score [15]. The presence of CAC was defined as an Agatston score of >0.

### Statistical analysis

Continuous variables with a normal distribution were expressed as the mean ± SD, whereas continuous variables with a skewed distribution were presented as median (interquartile range) and were log transformed for analysis. Intergroup comparisons were performed using ANOVA tests. Categorical variables with percentages were compared using Chi square tests. The relationships between CAC and various clinical parameters were examined using Pearson’s correlation. The relationship between the TyG index, HOMA-IR and the presence of CAC was analyzed using logistic regression analysis while controlling for potential confounders. Covariates in the multivariate model, which were chosen for their clinical importance, as well as statistical significance, were age, sex, SBP, BMI, LDL-C, HDL-C, smoking status, alcohol intake, and exercise habits. The area under the curve (AUC) of the receiver operating characteristics (ROC) curve and a 95% confidence interval (CI) were calculated to compare the predictive power of the TyG index and HOMA-IR for CAC. Subgroup analysis was performed after categorizing the subjects according to age, sex, BMI, BP, and LDL-C, and the odds ratio (OR) was compared between the highest and lowest quartiles of the TyG indices. All statistical analyses were performed using the SPSS statistical package, version 20.0 (SPSS, Inc., Chicago, IL, USA) and MedCalc (MedCalc software, Olstead, Belgium). *P* values of <0.05 were considered statistically significant.

## Results

Table 1 shows the clinical and biochemical characteristics of the enrolled subjects. The subjects were stratified into four groups based on their TyG index levels. Significant differences in metabolic parameters were observed among the groups. Age, SBP, DBP, BMI, FPG levels, TC levels, TG levels, LDL-C levels, insulin levels, and HOMA-IR increased and HDL-C levels decreased with an increase in the TyG index. In addition, significant differences in smoking status and alcohol intake were observed among the groups. Exercise habits were not found to be significant according to the TyG index.

**Table 1:** Clinical characteristics of subjects based on the TyG index

	Quartiles of the TyG index				*P*
	I (lowest)	II	III	IV (highest)	
N	1080	1083	1078	1078	
Age (years)	50.39 ± 9.86	52.77 ± 8.95	54.08 ± 8.87	56.36 ± 9.35	<0.01
Sex (M/F)	486/594	555/528	632/446	651/427	
SBP (mmHg)	118.35 ± 12.34	121.39 ± 12.16	125.49 ± 11.02	126.89 ± 11.28	<0.01
DBP (mmHg)	73.15 ± 7.15	75.17 ± 7.26	76.90 ± 8.13	78.43 ± 8.74	<0.01
BMI (kg/m^2^)	22.16 ± 2.81	23.26 ± 3.11	23.96 ± 2.88	24.97 ± 2.85	<0.01
FPG (mg/dL)	86.42 ± 9.23	92.79 ± 9.39	96.37 ± 9.74	98.87 ± 9.89	<0.01
TC (mg/dL)	184.55 ± 33.41	193.79 ± 33.68	195.99 ± 35.07	205.77 ± 35.03	<0.01
TG (mg/dL)	55 (48–62)	80 (73–89)	112 (100–124)	185 (156–230)	<0.01
LDL-C (mg/dL)	110.45 ± 29.65	121.94 ± 30.11	124.59 ± 31.50	125.76 ± 31.49	<0.01
HDL-C (mg/dL)	58.59 ± 12.88	53.05 ± 12.13	47.56 ± 10.72	42.77 ± 8.74	<0.01
TyG index	7.75 ± 0.21	8.22 ± 0.11	8.59 ± 0.11	9.15 ± 0.27	<0.01
Insulin (µU/mL)	2.95 (2.13–4.5)	4.0 (2.8–5.8)	4.6 (3.4–6.5)	5.5 (3.8–7.6)	<0.01
HOMA-IR	0.64 (0.44–0.99)	0.90 (0.64–1.33)	1.09 (0.78–1.57)	1.34 (0.88–1.87)	<0.01
Alcohol consumption (%)	92 (8.5)	95 (8.8)	120 (11.1)	201 (18.6)	0.03
Regular exercise (%)	129 (11.9)	148 (13.7)	128 (11.9)	101 (9.4)	0.12
Smoking (%)	329 (30.5)	354 (32.7)	367 (34.0)	393 (36.5)	<0.01

Data are represented as the mean ± SD, number (percentage), or median (interquartile range)

*SBP* systolic blood pressure, *DBP* diastolic blood pressure, *BMI* body mass index, *FPG* fasting plasma glucose, *TC* total cholesterol, *TG* triglyceride, *LDL-C* low-density lipoprotein cholesterol, *HDL-C* high-density lipoprotein cholesterol, *HOMA-IR* homeostasis model assessment of insulin resistance

**Fig. 1:**
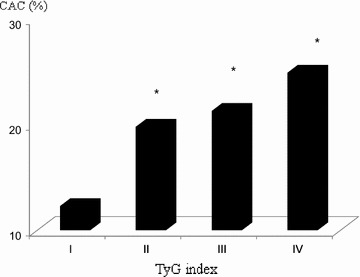
Prevalence of subjects with CAC based on the TyG index

In total, 4319 subjects were included. Of these, 3474 did not exhibit coronary artery calcification (CACS = 0), and the remaining 845 exhibited some evidence of CAC (CACS > 0, 19.6%). The prevalence of CAC and the mean CACS significantly increased with an increase in the TyG index (Table 2; Fig. 1).

**Table 2:** Prevalence of subjects with CAC and means of ln(CACS + 1) based on the TyG index

	Quartiles of TyG index				*P*
	I (lowest)	II	III	IV (highest)	
N	1080	1083	1078	1078	
ln(CACS + 1)	0.49 ± 0.04	0.82 ± 0.05	0.95 ± 0.06	1.14 ± 0.06	<0.01
CACS > 0, N (%)	133 (12.3)	214 (19.8)	230 (21.3)	268 (24.9)	<0.01

Data are represented as the mean ± SE or as a number (percentage)

*TyG* triglyceride glucose index, *CAC* coronary artery calcification, *CACS* coronary artery calcification score

Pearson correlation analysis was performed to examine the relationship between log-transformed CACS and various clinical parameters (Table 3). CAC showed significant correlations with the TyG index and HOMA-IR, and the correlation between the TyG index and CAC was better than the correlation between HOMA-IR and CAC.

**Table 3:** Correlations between CAC [ln(CACS + 1)] and risk factors

	*R*	*P*
Age	0.357	<0.01
Sex (male)	0.299	<0.01
SBP	0.161	<0.01
DBP	0.160	<0.01
BMI	0.116	<0.01
FPG	0.145	<0.01
TC	0.017	0.30
TG	0.109	<0.01
LDL-C	0.032	0.06
HDL-C	−0.105	<0.01
TyG index	0.138	<0.01
Insulin	0.098	<0.01
HOMA-IR	0.119	<0.01
Alcohol consumption	0.011	0.51
Regular exercise	0.026	0.21
Smoking	0.039	0.04

Continuous variables with skewed distributions (TG, insulin, HOMA-IR) were log transformed for analysis

*SBP* systolic blood pressure, *DBP* diastolic blood pressure, *BMI* body mass index, *FPG* fasting plasma glucose, *TC* total cholesterol, *TG* triglyceride, *LDL-C* low-density lipoprotein cholesterol, *HDL-C* high-density lipoprotein cholesterol, *TyG* triglyceride glucose index, *HOMA-IR* homeostasis model assessment of insulin resistance

The relationship between the TyG index and the presence of CAC (CACS > 0) was explored by categorizing the TyG index into quartiles using the first quartile as the reference (Table 4). An unadjusted multivariate logistic regression analysis, with I set as a reference, revealed that the TyG indices for II, III and IV had increased OR for the presence of CAC in all subjects (Model 1). After adjusting for confounding variables, including age, sex, SBP, BMI, LDL-C levels, HDL-C levels, smoking status, alcohol, and exercise habit, these relationships remained significant (*P* for trend < 0.05) (Model 3).

**Table 4:** OR for CAC based on the TyG index and HOMA-IR

	OR (95% CI)				*P* for trend
	I (lowest)	II	III	IV (highest)	
TyG index					
Model 1	1.00	1.74 (1.37–2.21)	2.02 (1.59–2.56)	2.73 (2.17–3.41)	<0.01
Model 2	1.00	1.30 (0.98–1.97)	1.47 (0.98–2.21)	2.18 (1.46–3.20)	<0.01
Model 3	1.00	1.18 (0.75–1.85)	1.28 (0.85–1.99)	1.95 (1.23–3.11)	0.01
HOMA-IR					
Model 1	1.00	1.70 (1.20–2.40)	2.38 (1.70–3.34)	2.56 (1.82–3.61)	<0.01
Model 2	1.00	1.47 (1.01–2.14)	1.76 (1.24–2.60)	1.87 (1.29–2.73)	<0.01
Model 3	1.00	1.36 (0.94–1.97)	1.62 (1.12–2.34)	1.64 (1.12–2.40)	0.04

Model 1: unadjusted

Model 2: adjusted for age and sex

Model 3: adjusted for age, sex, SBP, BMI, LDL-C levels, HDL-C levels, smoking status, alcohol, and exercise habits

*TyG* triglyceride glucose index, *HOMA-IR* homeostasis model assessment of insulin resistance, *CAC* coronary artery calcification, *CI* confidence intervals, *OR* odds ratio

We further evaluated the relationship between the HOMA-IR and the presence of CAC by categorizing HOMA-IR into quartiles using the first quartile as the reference (Table 4). The higher HOMA-IR quartile was significantly associated with the presence of CAC, and these relationships remained significant even after adjusting for confounding variables. After adjustment for multiple risk factors, the OR (95% CI) for having CAC of HOMA-IR in the highest quartile was 1.64 (1.12–2.40), whereas the OR of the TyG index in the highest quartile was 1.95 (1.23–3.11). The AUC (95% CI) of the ROC curve for the TyG index at 0.629 (0.598–0.660) was significantly higher than that of the HOMA-IR at 0.585 (0.554–0.616) (P = 0.016). These results suggest that the TyG index is better than HOMA-IR in predicting CAC.

Adjusted ORs of CAC subgroups were significant regardless of age, sex, BMI, BP, or LDL-C levels. In addition, subjects who were ≥50 years old, male, obese (BMI ≥ 25 kg/m^2^), had high BP (≥120/80 mmHg), and had high LDL-C (≥130 mg/dL) showed higher ORs compared with those without these risk factors (Additional file 1: Table S1).

## Discussion

In this study, we investigated the relationship between the TyG index and CAC in healthy Korean adults and found that the prevalence of CAC was significantly associated with the TyG index, and was independent of conventional cardiovascular risk factors. To the best of our knowledge, this is the first study to investigate the relationship between the TyG index and the presence of early coronary artery atherosclerosis in healthy adults. Furthermore, the TyG index showed higher predictability for CAC than did HOMA-IR.

IR has been proposed as an important cause of several metabolic disease [16–18] and CVD [4–6]. The gold standard technique for assessing insulin sensitivity is the hyperinsulinemic euglycemic clamp test [19], but this method is invasive, laborious, and expensive, and it can only be used for research purposes. Therefore, HOMA-IR was developed as a more convenient method for measuring IR and is widely used in clinical settings [20, 21]. Recently, some studies have shown that the TyG index is correlated with IR, as assessed using the hyperinsulinemic euglycemic clamp test and HOMA-IR, and the TyG index has been considered to be a surrogate marker of IR [7–9, 22, 23]. In addition, some studies have shown that the predictive value of the TyG index is better than that of HOMA-IR [10, 24].

Few reports on the relationship between the TyG index and CVD events exist, and these are controversial. In a recent study, Sanchez-Inigo et al. reported that the TyG index might be useful to identify individuals at high risk of a CVD event [13], whereas another study has shown that the TyG index does not predict CVD events [12]. Furthermore, to date, only two studies have examined the relationship between the TyG index and subclinical atherosclerosis. Irace et al. reported that the TyG index is significantly associated with carotid artery atherosclerosis after adjustment for traditional cardiovascular risk factors [25]. Recently, Lee et al. showed an independent relationship between the TyG index and significant coronary artery stenosis in patients with type 2 diabetes [26], but the sample size was small (n = 888) and only subjects with type 2 diabetes were included.

In the present study, we demonstrated that the TyG index was independently associated with the presence of CAC in healthy adults. Compared with HOMA-IR, the TyG index is better for predicting subclinical atherosclerosis, and this result corroborates the previous finding that the TyG index showed a stronger association with carotid atherosclerosis than did HOMA-IR [25].

Although the mechanism underlying the relationship between the TyG index and CAC has not been clarified, it may be linked to IR. Many studies have indicated that IR leads to inflammation, altered coagulation and atherosclerosis [27–30]. Furthermore, an independent association between IR and CAC has been reported [31–33]. Both the TyG index and HOMA-IR are well known representative markers of IR, and they are closely related to each other. However, our findings showed that the TyG index was better associated with the presence of coronary artery atherosclerosis than was HOMA-IR, and this result may be explained by the fact that the two indices reflect different aspects of IR. The TyG index reflects IR mainly in the muscle [34–36], and HOMA-IR reflects IR mainly in the liver [37, 38], which may have caused the difference. Peripheral insulin resistance may be a useful marker of coronary artery atherosclerosis [25].

Our study also had some limitations. First, this was a cross-sectional observational study that could not definitively establish causality. Thus, the precise causal relationship between the TyG index and CAC remains controversial. Second, our study population was relatively small. Third, we did not compare the TyG index using the hyperinsulinemic euglycemic clamp test, the gold standard method for measuring IR. Fourth, we could not record nutritional data. Thus, we could not adjust for nutritional habits, which can affect blood triglyceride levels. Lastly, the participants in the current study were enrolled at one health promotion center, and thus the generalizability of the results may be limited.

## Conclusions

The TyG index was significantly associated with the prevalence of CAC, and this relationship remained significant after adjusting for conventional factors. Moreover, the TyG index was more independently associated with the presence of coronary artery atherosclerosis than was HOMA-IR in healthy Korean adults. The TyG index might be a useful marker of atherosclerosis, reflecting cardiovascular risk. Further prospective large-scale studies are required to clarify the mechanisms of this relationship.

## Additional file

**Additional file 1: Table S1.** Adjusted odds ratio (95% CI) of subgroup for coronary artery calcification (CACS > 0).

## Abbreviations

TyG: triglyceride glucose
CAC: coronary artery calcification
CACS: coronary artery calcium score
CT: computed tomography
CVD: cardiovascular disease
IR: insulin resistance
BMI: body mass index
BP: blood pressure
FPG: fasting plasma glucose
TC: total cholesterol
HDL-C: high-density lipoprotein cholesterol
LDL-C: low-density lipoprotein cholesterol
HOMA-IR: homeostasis model assessment of insulin resistance
TG: triglycerides
OR: odds ratio
SBP: systolic blood pressure
DBP: diastolic blood pressure
