# Supplementary material for: Relationship between the triglyceride glucose index and coronary artery calcification in Korean adults
Source: Cardiovasc Diabetol. 2017 Aug 23;16:108. doi: 10.1186/s12933-017-0589-4 (PMC5568209; doi:10.1186/s12933-017-0589-4)
Supplement: Supplementary file 1 — Additional file 1: Table S1. Adjusted odds ratio (95% CI) of subgroup for coronary artery calcification (CACS > 0). [file 12933_2017_589_MOESM1_ESM.docx]

**Additional file 1: Table S1. Adjusted odds ratio (95% CI) of subgroup for coronary artery calcification (CACS > 0)**

|  | Odds Ratio (95% CI) | P value |
| --- | --- | --- |
| Age |  |  |
| < 50 years | 1.68 (1.19-2.37) | 0.03 |
| > 50 years | 1.92 (1.14-3.23) | <0.01 |
| Sex |  |  |
| Male | 1.79 (1.30-5.45) | <0.01 |
| Female | 1.34 (1.09-1.80) | <0.01 |
| BMI |  |  |
| < 25 kg/m^2^ | 1.51 (1.06-2.17) | 0.01 |
| > 25 kg/m^2^ | 1.96 (1.25-3.06) | <0.01 |
| BP |  |  |
| < 120/80 mmHg | 1.25 (1.10-1.78) | 0.03 |
| > 120/80 mmHg | 2.16 (1.53-3.05) | <0.01 |
| LDL |  |  |
| < 130 mg/dL | 1.61 (0.89-2.89) | 0.04 |
| > 130 mg/dL | 2.57 (1.27-5.18) | 0.01 |

BMI, body mass index; BP, blood pressure; LDL-C, low-density lipoprotein cholesterol. Odds ratio of the highest quartile of TyG index was showed in comparison with the lowest quartile of TyG index.
